# Effects of *Aspergillus niger* Infection on the Quality of Jujube and Ochratoxin A Cumulative Effect

**DOI:** 10.3390/toxins15070406

**Published:** 2023-06-21

**Authors:** Xueyan Xin, Mina Nan, Yang Bi, Huali Xue, Yuan Zhang, Jiajie Wang, Zhiwei Lu

**Affiliations:** 1 College of Science, Gansu Agricultural University, Lanzhou 730070, China; xinxy@st.gsau.edu.cn (X.X.); wangjj@st.gsau.edc.cn (J.W.); luzw@st.gsau.edc.cn (Z.L.); 2 Basic Experiment Teaching Center, Gansu Agricultural University, Lanzhou 730070, China; zhangyuan@gasu.edc.cn; 3 College of Food Science and Engineering, Gansu Agricultural University, Lanzhou 730070, China; biyang@gsau.edu.cn

**Keywords:** jujube, quality, *Aspergillus niger* (H1), ochratoxin A

## Abstract

The jujube is one of the most popular fruits in China because of its delicious taste and high nutritional value. It has a long history of usage as an important food or traditional medicine. However, the jujube is easily infected by fungi, which causes economic losses and threatens human health. When the jujube was infected by *Aspergillus niger* (H1), the changes in nutritional qualities were determined, such as the content of total acid, vitamin C, reducing sugar, etc. In addition, the ability of *A. niger* (H1) to produce ochratoxin A (OTA) in different inoculation times and culture media was evaluated, and the content of OTA in jujubes was also analyzed. After jujubes were infected by A. *niger* (H1), the total acid, and vitamin C contents increased, while the total phenol content decreased, and the reducing sugar content increased after an initial decrease. Although *A. niger* (H1) infection caused the jujubes to rot and affected its quality, OTA had not been detected. This research provides a theoretical foundation for maximizing edible safety and evaluating the losses caused by fungal disease in jujubes.

## 1. Introduction

The jujube (*Ziziphus zizyphus*) is one of the oldest cultivated fruit trees around the world, and is the most important species in the large cosmopolitan family *Rhamnaceae* in terms of its economic, ecological, and social importance [1]. The jujube is referred to as “grain on trees,” and has been cultivated for over 4000 years [2]. It has been introduced into at least 48 countries since it was first introduced into countries such as Korea and Japan 2000 years ago [3,4]. In China, jujube is considered an important health food due to its high nutritional value, which integrates three major functions: food, tonic, and medicine. The jujube is an excellent source of proteins, carbohydrates, minerals, vitamins, polyphenols, and other essential nutrients needed for optimal health [5]. It is a famous, commonly used traditional Chinese medicine that is used in 50% of Chinese herbal medicine prescriptions [6]. In recent years, with the changes in the planting ecological environment, low levels of agricultural technology, and the increase in susceptible hosts for pathogens, the diseases in jujubes have been increasing year by year. The jujube is easily infected by fungi such as *A. niger*, *Aspergillus flavus*, and *Alternaria*, and produces mycotoxins during growth and maturation [7,8]. According to the report, there are 38 diseases/disease complexes that infect jujubes during pre-and post-harvest stages [9]. Moreover, some of these diseases will cause worsening quality, rendering jujubes non-edible. In view of reducing economic losses and protecting consumer health, it is essential to study the quality deterioration caused by jujube diseases.

*A. niger* is a filamentous ascomycete fungus, and one of the most usually used industrial *Aspergillus* species [10]. It is most widely known for its widespread distribution, such as grain, plant products, and soil. Infections due to *Aspergillus* species can lead to serious morbidity and mortality of fruits and vegetables. Moreover, at appropriate temperatures and humidity, *A. niger* multiplies easily and produces secondary metabolites, which will adversely affect the quality and flavor of fruits [11]. The jujube fruit is inevitably subjected to different types of damage during growth, harvest, storage, transportation, and processing, which further exacerbates *A. niger* infections. As one of the most significant pathogens, *A. niger* severely influences the quality and marketing value of jujubes. Chen et al. found that *A. niger*, which has the highest pathogenicity and isolation frequency, is one of the main pathogens causing jujube mildew and rot in Xinjiang, China [12]. Zhang et al. investigated the main fungal diseases of *Zizyphus mauritiana* Lam in the Yunnan Province of China, and found that the pathogenic fungi of jujubes involved nine genera, among which *A. niger* was a common one [13]. Guo et al. studied the biology of the Yuancui jujubes infected by *Aspergillus niger* TL-10 and its control after harvest, and the results showed that *A. niger* TL-10 could infect injured jujube, and the infection rate reached 100% at an inoculum concentration of above 1 × 10^2^ spores/mL [14]. Other studies have found *A. niger* is one of the main dominant pathogens causing the disease of dried ash jujube fruit [15]. However, to date, there is no research about the effect of *A. niger* on jujube quality.

Ochratoxin A (OTA) is a toxic secondary fungal metabolite produced mainly by some species of *Aspergillus* and *Penicillium* fungi in different hosts [16]. It has been reported that the toxicity of OTA is related to the occurrence of many diseases in animals and humans, such as nephrotoxicity, hepatotoxicity, teratogenicity, immunotoxicity, and neurotoxicity [17]. In recent years, *A. niger* has been isolated from different hosts, such as cereals, coffee, grapes, and jujubes, and its metabolite OTA has been detected [18]. OTA was detected in 121 dried fruit samples from Iran by an enzyme-linked immunosorbent assay (ELISA) technique, and found that the incidence of OTA was 10% in jujube [19]. A total of 117 dried fruit samples were analyzed for both toxigenic fungi and the presence of ochratoxin A, and the OTA found in jujubes did not exceed 5 µg/kg [20]. A total of 48 jujube samples of different local date varieties were collected from Tunisian markets, and OTA was detected in 38% (mean 1.26 μg/kg) [21]. The levels of OTA in 20 different varieties of jujubes were detected, and it was found that all samples contained low-content OTA [22]. Therefore, it is necessary to investigate the effects of *A. niger* infection on the nutritional quality, and the OTA accumulation in jujubes.

Fungal diseases and mycotoxin contamination have been serious problems in the jujube industry. Therefore, it is very important to minimize the potential risks to humans and the economic losses. The present investigation was designed to find out whether *A. niger* infection caused mycotoxin contamination and quality loss in jujubes. The nutritional quality of jujubes were tested before and after *A. niger* (H1) infection, including total acid, reducing sugar, total phenol, vitamin C, weight loss, degree of browning, color value, and diameter of the lesion. Meanwhile, the accumulation of OTA was also determined, in order to assess the OTA-producing potential of *A. niger* (H1) in jujubes. This research is of great significance to improve the quality of jujubes and to maximize the edible safety of jujubes.

## 2. Results

### 2.1. Effect of A. niger (H1) Infection on the Quality of Jujube

#### 2.1.1. Effect of *A. niger* (H1) Infection on the Lesion Diameter

**Figure 1:**
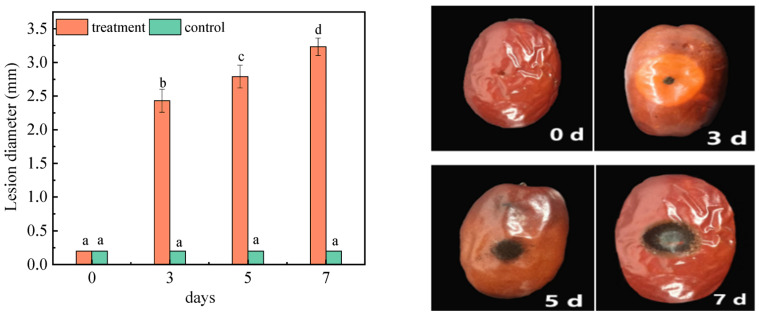
Changes in the lesion diameters of jujube before and after infection with *A. niger* (H1). All data are expressed as means ± standard deviation of triplicate samples. Different lower-case letters indicate significant differences according to Duncan’s test (*p* < 0.05). Vertical bars represent the standard errors of the means (*n* = 3).

The lesion diameter of the jujubes was determined 0, 3, 5, and 7 days after inoculation (DAI) and shown in Figure 1. The results demonstrated that there was no significant change in the diameter of the lesions (0.2 cm) in the control group. On the first day of *A. niger* (H1) infection, the lesion diameter was measured as 0.2 cm, while on the 7 DAI, the lesion diameter expanded to 3.23 cm. The diameter of the lesions showed a significant change with the extension of inoculation time. At the early stage of infection (2 DAI), although lesions with visible black spores around the wounded hole were small, the lesions became soft and watery. In the late stage of storage, decay of the tissues developed with time, lesions kept expanding, and more black spores were found around inoculation holes. This indicated that *A. niger* (H1) inoculation can cause rot, and can increase the diameter of the lesion in jujubes. Moreover, *A. niger* is a highly resistant fungi that infects early fruits, leading to the post-harvest spoilage of fruits. *A. niger* infects the fruit mainly by producing stratum cuticle and cell-wall-degrading enzymes, toxins and detoxification resistance compounds [23]. Li et al. studied the pathogenic law of *A. niger* on red globe grapes, the results showed that the injury was most obvious when the wound was 3–4 mm, the inoculated pathogen amounted for 10^5^ spores/mL [24]. It can be found that the lesion diameter of *A. niger*-infected grapes is larger than that of jujubes. The reason is that *A. niger* (H1) was isolated from grapes, and the nutritional conditions of grapes are more suitable for *A. niger* growth, so the harm of *A. niger* to grapes and the risk of mycotoxin contamination are both more serious than those of red dates.

#### 2.1.2. Effect of *A. niger* (H1) Infection on Weight Loss

As one of the main components of jujubes, water directly affects the life activities of microorganisms and the rate of chemical reaction in jujubes. Weight loss is closely related to water content. A faster weight loss rate led to serious moisture loss, and affected the taste of jujubes. The weight loss of jujubes was calculated after 0, 3, 5, and 7 days of inoculation, and is shown in Table 1. There was a significant change in weight loss before and after infections, at 7 DAI. The results showed that a large amount of water was lost, which makes the taste of jujubes worse. *A. niger* (H1) forms a mycelial network in jujube incubation. Because the hypha contacts water in the air, the number of water-stable aggregates will increase. Therefore, there was no significant loss of water after infection. The contact between hyphae and moisture in the air increased the number of water-stable large aggregates in jujubes, so there was no significant loss of water in the jujubes during the inoculation [25]. The most obvious phenomenon caused by continuous transpiration and dehydration of fruits during storage is weight loss. Fruit weight loss can lead to loss of surface gloss and even commodity value. It was found that the weight loss rate of grapes decreased after inoculation with *A. niger*, and H_2_S intermittent fumigation could inhibit this phenomenon [26].

#### 2.1.3. Effect of *A. niger* (H1) Infection on Reducing Sugar

**Figure 2:**
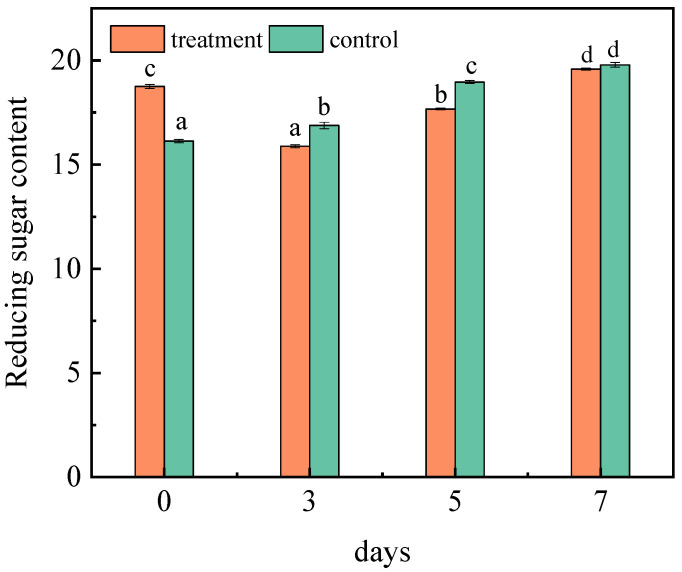
Changes in the reducing sugar content in jujubes before and after infection with *A. niger* (H1). All data are expressed as means ± standard deviation of triplicate samples. Different lower-case letters indicate significant differences according to Duncan’s test (*p* < 0.05). Vertical bars represent the standard errors of the means (*n* = 3).

It is reported that reducing sugar content accounts for more than 70% of the total sugar content of jujubes [27]. Figure 2 showed the changes in the reducing sugar content. Under the same experimental conditions, the content of the reducing sugar in the control group changed significantly, and increased to 19.78% on the seventh day. Due to the extension of storage time, water evaporation and microbial decomposition will occur in jujubes, leading to an increase in the reducing sugar content. After receiving the inoculation, the reducing sugar content started to decline on the third and fifth days. A previous study showed that the sugar content in the host also decreased because the fungi consumed carbon sources to meet its growth needs [28]. Therefore, *A. niger* (H1) will preferentially use carbohydrates to promote its development and reproduction in the early stage of inoculation [29]. Probably because, as the fungi propagated further, the consumption of carbohydrates increased, resulting in a decrease in reducing sugar content. However, on the seventh day, the content was 19.58%. That is because, in the medium and later growth stages of *A. niger* (H1), it began to consume protein and other nutritious substances for its energy metabolism. Thereafter, the oxidation rate of reducing sugar or the rate of polysaccharide decomposition into small molecules will slow down and, finally, the content of reducing sugar will be maintained [30]. In addition, according to Lei’s research, symbiotic fungi also increase the content of glucose and fructose to varying degrees, which leads to further increases in sugar content [31].

#### 2.1.4. Effect of *A. niger* (H1) Infection on Total Acid

**Figure 3:**
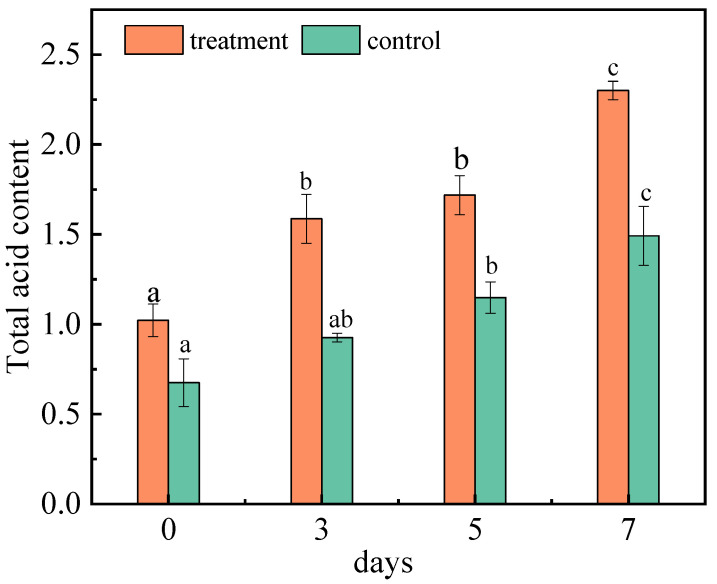
Changes in the total acid content of jujubes before and after infection with *A. niger* (H1). All data are expressed as means ± standard deviation of triplicate samples. Different lower-case letters indicate significant differences according to Duncan’s test (*p* < 0.05). Vertical bars represent the standard errors of the means (*n* = 3).

Acids are important metabolites in fruits, which have important effects on fruit quality, taste, nutritional value, and freshness. The changes in the acid content also reflect the consumption and metabolism of nutrients in jujubes. The total acid content of jujubes was determined respectively at 0, 3, 5, and 7 days (Figure 3). With the continuous extension of inoculation days, the total acid content of the inoculated group and control group both increased. On the seventh day, it reached 2.30% and 1.50%, respectively. *A. niger* (H1) has a high level of activity in producing acid protease, which is conducive to improving the production rate of amino acids [32]. Therefore, after inoculation of jujubes with *A. niger* (H1), the increase in the amino acid content led to a significant increase in the total acid content. Fan et al. studied the changes in the nutrients in red jujubes after being infected with black spot disease; the results showed that the content of total acid was significantly reduced, which is inconsistent with this research [33]. The principal reason for this is that there are significant differences in the nutritional requirements of the host between the growth of *Alternaria* and *Aspergillus*. However, after apples were inoculated with *Valsa ceratosperma* in 0–7 days, salicylic acid, pcoumaric acid, cinnamic acid and chlorogenic acid increased significantly [34]. The plant resistance induced by fungi may be the result of the protein expression of salicylic-acid-mediated acidic pathogen, and the content of acids related to the synthesis of salicylic acid increased accordingly [35].

#### 2.1.5. Effect of *A. niger* (H1) Infection on Vitamin C

**Figure 4:**
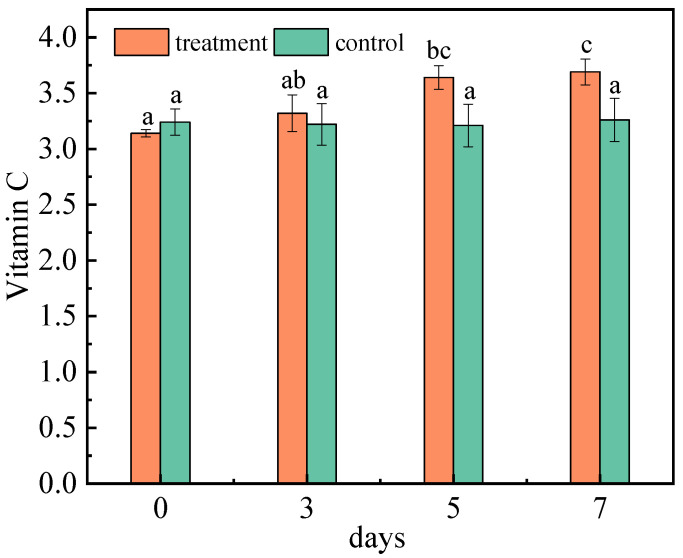
Changes in vitamin C content of jujubes before and after infection with *A. niger* (H1). All data are expressed as means ± standard deviation of triplicate samples. Different lower-case letters indicate significant differences according to Duncan’s test (*p* < 0.05). Vertical bars represent the standard errors of the means (*n* = 3).

Vitamin C (VC) is one of the most important nutrients in the jujube. As a reducing substance, VC is very easy to oxidize. In addition, it is not only an important substance to protect membrane lipids, but also a momentous antioxidant substance for scavenging active oxygen in the fruit. Therefore, it can be used as an indicator of anti-aging and stress resistance [36]. The VC content was determined at 0–7 DAI (Figure 4). The content of vitamin C in the control group remained nearly constant. The main oxidase, such as ascorbic acid oxidase, can maintain the activity for 40 min below 50 °C and oxidize VC to produce dehydroascorbic acid [37]. However, dehydroascorbic acid is quickly reduced to VC by dehydroascorbic acid reductase, which compensates for partially decomposed VC [38]. The VC content increased after the infection with *A. niger* (H1), and reached 3.69 mg/g on the seventh day. Jujubes contain a large number of pectin substances, but pectinase produced by filamentous fungi, especially *Aspergillus niger*, can decompose pectin substances in jujubes, leading to an increase in VC content [39]. Additionally, VC is a kind of water-soluble and acidic polyhydroxy compound with the properties of acid. After inoculation with *A. niger* (H1), the total acid content of the jujubes increased, which is beneficial for the stability and accumulation of VC [40]. Moreover, jujubes contain an ascorbic acid enzyme that destroys vitamins; the longer the storage time, the more vitamins are destroyed, and the less the content. Although it is uncertain whether *A. niger* destroyed the release of ascorbic acid enzyme in jujubes, it significantly delayed the release of vitamin C [41].

#### 2.1.6. Effect of *A. niger* (H1) Infection on Browning Index and Total Phenol

**Figure 5:**
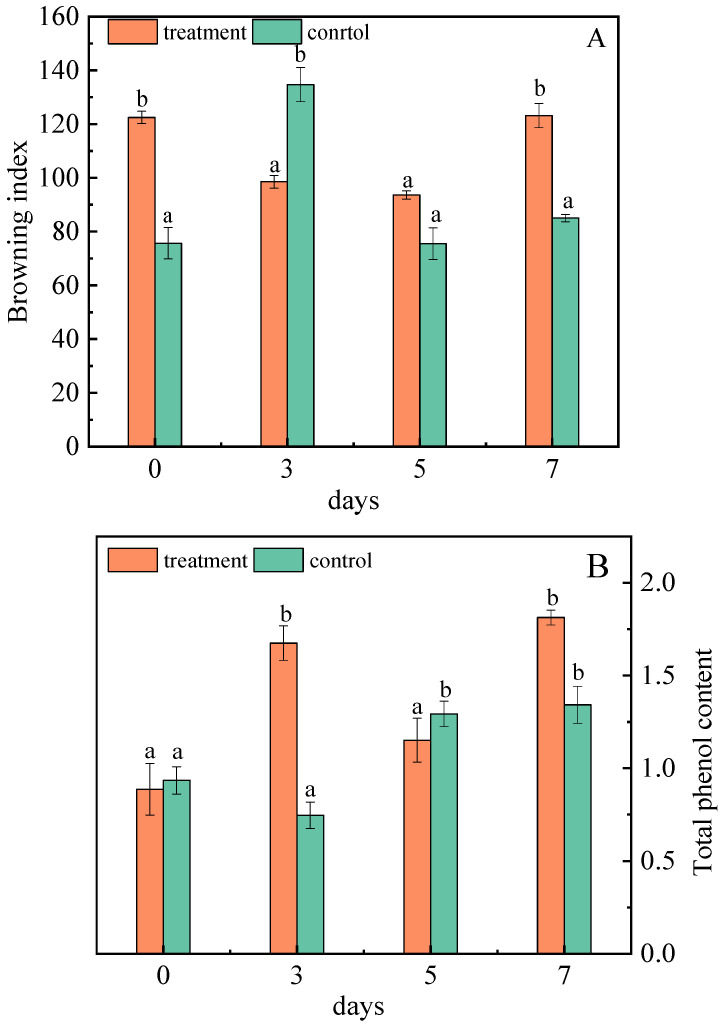
Changes in the browning degree (**A**) and total phenol (**B**) of jujubes before and after infection with *A. niger* (H1). All data are expressed as means ± standard deviation of triplicate samples. Different lower-case letters indicate significant differences according to Duncan’s test (*p* < 0.05). Vertical bars represent the standard errors of the means (*n* = 3).

The browning of the jujube reflects its color, sensory quality, and commodity value. The browning index decreased in the third and fifth days after inoculation, but showed an increaseing trend on the seventh day. However, on the third day of inoculation, the browning index was lower than that of the control group (Figure 5A). In control group, this is due to phenols that are oxidized to form quinones substances, and quinones that form brown substances through self-polymerization or polymerization with other compounds contained in the -NH_2_, -SH groups, leading to browning, deepening of the color of the fruit flesh, and increasing in the degree of browning [42]. Glucose and fructose in jujubes can not only participate in the Maillard reaction, producing dark brown substances, but are also an important carbon source for the growth of *A. niger* (H1), which can produce black spores, secrete melanin, and increase the degree of browning [43].

After infection with *A. niger* (H1), the total phenol content of the jujubes changed significantly. On the seventh day, the total phenol content was 1.81 mg/g, 35.07% higher than that of the control (Figure 5B). Liao et al. also suggested that *A. niger* was more capable of increasing polyphenol content than the others in the study [44], which is consistent with this research. The complex enzymes (cellulose hydrolase, lignin hydrolase, pectin hydrolase, etc.) produced by *A. niger* destroys the dense structure of the cell wall, which helps to expose the chemical bonds between phenolic substances and other substances. Then, cellulase breaks the covalent bond between polyphenol and cellulose, hemicellulose, lignin, etc., thus promoting the release of phenolic substances and increasing the content of total phenols [45].

#### 2.1.7. Effect of *A. niger* (H1) Infection on Color Value

**Figure 6:**
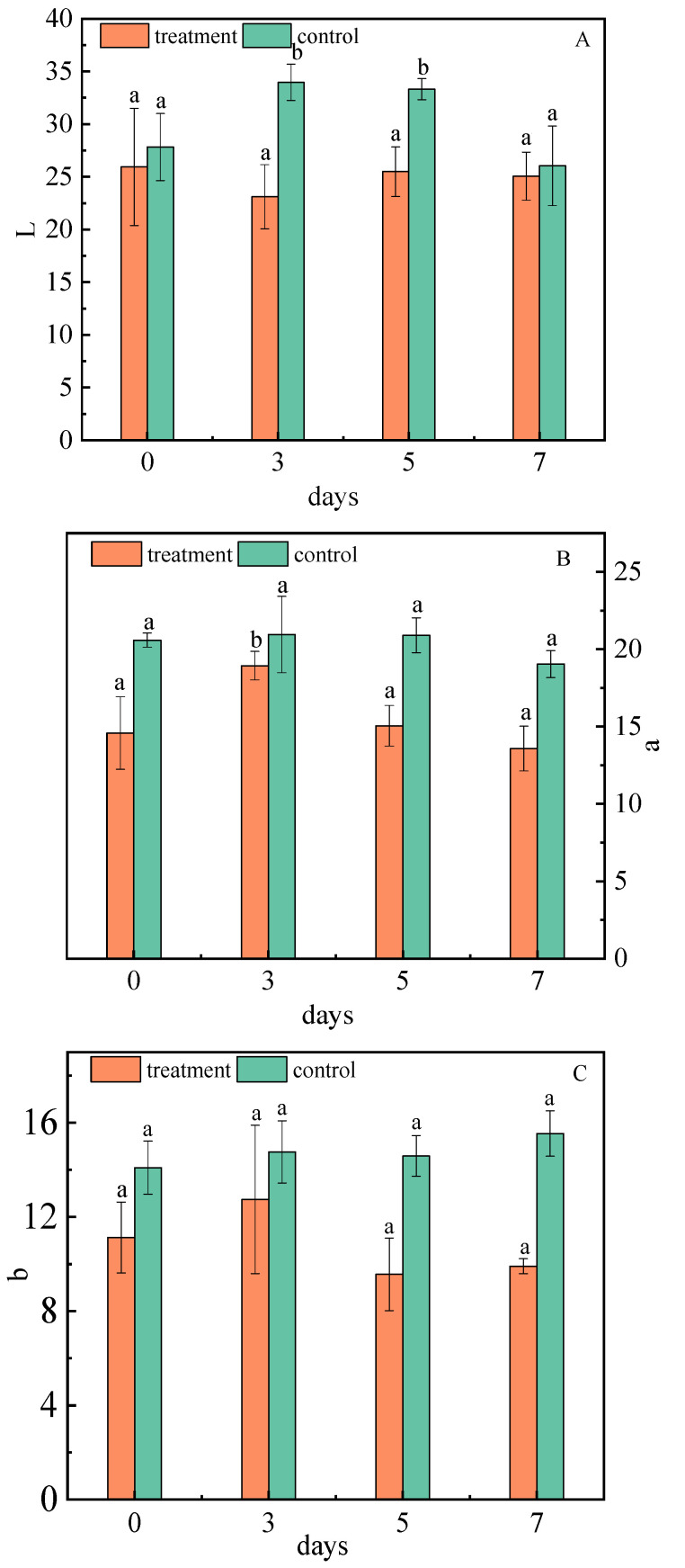
Changes of L* (**A**), a* (**B**), and b* (**C**) values of jujubes before and after infection with *A. niger* (H1). All data are expressed as means ± standard deviation of triplicate samples. Different lower-case letters indicate significant differences according to Duncan’s test (*p* < 0.05). Vertical bars represent the standard errors of the means (*n* = 3).

The Hunter L* (0—black, 100—white), a* (green to red), and b* (blue to yellow) values for the fruit skin color were determined [46]. The color values of the jujubes showed that the inoculation significantly decreased the lightness component of the skin. On the third day, the skin luminance was lower than 8.01% of the control (Figure 6A). This is because the non-enzymatic browning of phenols leads to a decrease in skin luminance, and the Maillard reaction of free amino acid and reducing sugar can also reduce the color value of jujubes [47]. Tian studied the effect of *A. niger* on the quality formation of Southern-route tea in the process of fermentation, and proposed that the L* color values of dry tea in *A. niger* treated samples has decreased [48].

After infection, the values of a* and b* first increased and then decreased, and were consistently lower than the control group. The value of a* showed a major difference on the fifth day, which was 5.85% lower than that of the control group (Figure 6B). The value of b* was different on the seventh day, which was 6.44% lower than that of the control group (Figure 6C). Cellulase produced by *A. niger* (H1) has strong activity which can change the permeability of the cell wall through hydrolysis, catalyze the decomposition of fruit skin tissue, and accelerate the dissolution of pigments [49,50]. Finally, the red and yellow intensity is reduced; that is, the a* and b* of the jujubes are decreased.

#### 2.1.8. Interaction Model between the Different Qualities and *A. niger*

**Figure 7:**
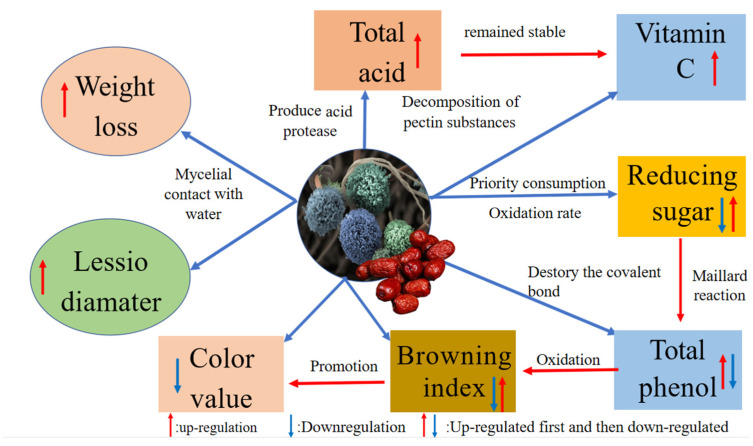
The proposed model of interaction between the different qualities and *A. niger*.

Fruit spoilage resulting from postharvest fungal pathogens is a global problem. *A. niger*, which is an important postharvest pathogen, has a high separation rate after harvest, and can cause fruit soft rot during storage. The experimental results mentioned above indicated the quality of jujubes changed significantly after infection with *A. niger*, and the proposed model of interaction between the different qualities and *A. niger* is shown in Figure 7. *A. niger* infects jujubes and forms a mycelial network, which contacts water in the air and increases the number of water-stable large aggregates in jujubes, causing the change in weight loss before and after infections [16]. Reducing sugars were first consumed as carbon sources for its growth, and then the oxidation rate of the reducing sugars and the polysaccharide decomposition rate were slowed down, showing a trend of first decreasing and then increasing [28,30]. *A. niger* produced complex enzymes that break the covalent bonds between polyphenol and components of plant cell walls, and release phenolic substances, which increased the content of total phenols [45]. The oxidation of phenolic substances form quinones and lead to browning [42]. The level of the occurrence and development of browning are reasons for the change in luminance. Additionally, the Maillard reaction in reducing sugars produce dark brown substances [43]. With the combined effects of the phenolic substances and the reducing sugars, the browning index of jujubes first decreased, then increased. The increase of amino acid and salicylic acid content led to an increase in the total acid content of jujubes after inoculation *A. niger* is helpful for the stability and accumulation of VC [32,40]. Thus, VC and total acid were both increased after *A. niger* inoculation.

### 2.2. Effect of A. niger (H1) Infection on OTA Content in Jujube

#### 2.2.1. Standard Curve of OTA

**Figure 8:**
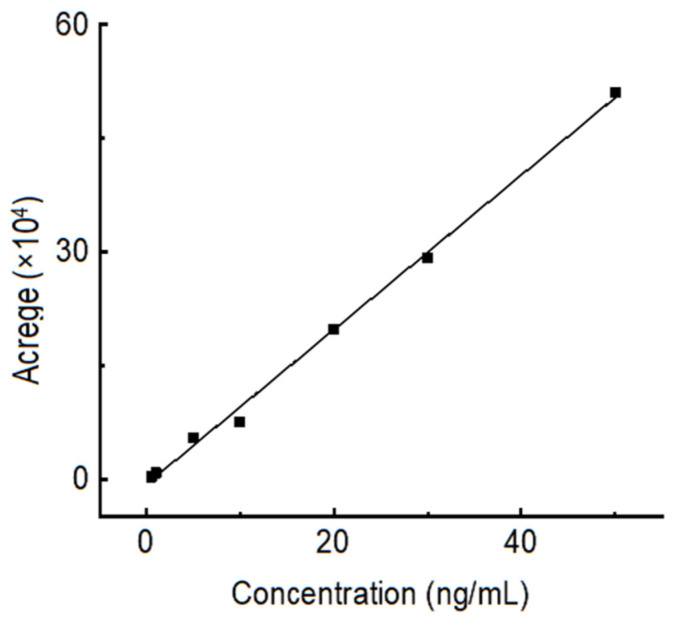
Standard curve of OTA with different concentrations, from 0.5 ng/mL to 50 ng/mL.

The standard curve of OTA with different concentrations, from 0.5 ng/mL to 50 ng/mL, is shown in Figure 8. The absorption peak areas (A) had a linear relationship with the concentration of OTA (C_OTA_), the linear regression equation of OTA was A = 0 + 31274.91C_OTA_ (ng/mL), and the linear correlation coefficient R^2^ was 0.9969.

#### 2.2.2. OTA Production of *A. niger* (H1) Strains in Different Media

**Figure 9:**
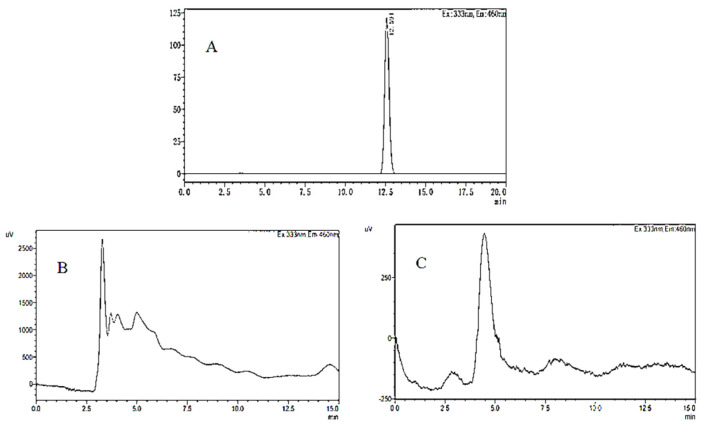
HPLC−FD chromatograms of OTA in OTA standard (**A**), YES medium containing jujube juice (**B**), and PDA medium containing jujube juice (**C**).

The chromatogram of the OTA standard solution is shown in Figure 9A. It can be seen from the figure that the chromatographic peak baseline is stable, the peak shape is symmetrical and clear, and the retention time is 12.5 min. OTA production of *A. niger* (H1) was determined in two different kinds of media: YES medium (Figure 9B) and PDA medium (Figure 9C). OTA in the YES and PDA media was extracted and analyzed. No chromatographic peak of OTA was observed with the same retention time in the chromatogram; that is, no OTA was detected in these media. However, according to some studies, *A. niger* strains can produce OTA in both YES and CYA media [51]. In addition, He et al. found that *A. niger* could also produce OTA after *A. niger* was inoculated on a coconut cream agar (CCA) [52]. In the present study, the initial media conditions have been changed by adding jujube juice, which affected the toxin occurrence of *A. niger* (H1). That meant *A. niger* (H1) could produce OTA under specific conditions, and the toxin-producing conditions are critical.

#### 2.2.3. OTA Accumulation in Jujubes after *A. niger* (H1) Infection

**Figure 10:**
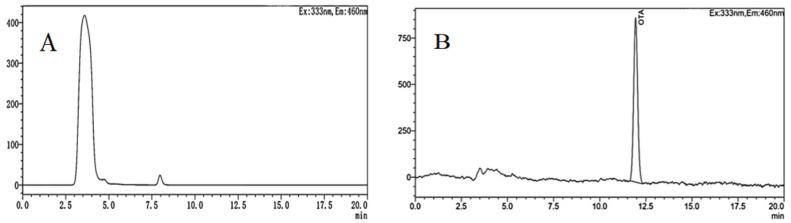
HPLC-FD chromatograms of OTA in jujubes (**A**) and grapes (**B**) infected by *A. niger* (H1).

The HPLC−FD chromatogram obtained from grapes infected by *A. niger* (H1) is shown in Figure 10. As can be seen, the concentration of OTA from the grapes was 3.030 ng/mL, and the retention time was 12 min, which was consistent with the retention time of the OTA standard. Compared with grapes, the chromatographic peak of OTA was not observed at the same retention time in jujubes. That is because *A. niger* (H1) was isolated from the grapes, and the nutritional conditions of jujubes were different from those of grapes, especially sugar, acid, water, etc. Furthermore, OTA-producing fungi are more likely to infect fruits with thinner skins, lower acidity, and higher sugar content and water activity [53]. Thus, when the *A. niger* (H1) infects the jujube, the nutritional conditions of the host directly affect the occurrence of OTA. In addition, a previous study found that *A. niger* can degrade OTA in the liquid YES medium [54]. Varga and co-workers have reported the degradation of ochratoxin A by some strains of *A. niger* [55]. In a similar study, Bejaoui et al. proved that *A. niger* can degrade 80% of OTA (2 mg/L) [56].

## 3. Conclusions

The nutrition quality changes of jujubes after 0–7 days of inoculation with *A. niger* (H1) were studied in this research. The results showed that the jujube would lose water after *A. niger* (H1) infection, which made the jujube skin shrink seriously. L*, a*, b*, and browning degrees decreased significantly, resulting in the deterioration of the surface gloss of the jujube. In addition, the content of VC and total acid increased continuously, and the content of reducing sugars first increased, then decreased. The OTA-producing ability of *A. niger* (H1) in different media was also analyzed, which indicated that no OTA was detected in the two different media and *A. niger* (H1)-infected jujubes. Based on these results, it can be concluded that *A. niger* (H1) had a negative impact on some quality of the jujubes, but it did not produce OTA during the infection process. Although more studies should be systematically conducted on other OTA-producing fungi in jujubes, it is not implying that there is a high risk of human exposure to OTA through the consumption of jujubes. However, in order to reduce the economic loss to the jujube industry, reduction and control the infection of fungi in agricultural and manufacturing practices is needed.

## 4. Materials and Methods

### 4.1. Chemical Reagents

The materials and reagents required for this experiment were as follows: jujubes were purchased from the market. *A. niger* (H1) was isolated from the wine-producing region in the Hexi Corridor, Gansu Province, and was provided by the College of Food Science and Engineering, Gansu Agricultural University, Lanzhou, China. Agar was purchased from Beijing Kulaibo Technology Co., Ltd., Beijing, China. YES medium was obtained from Thermo Fisher Scientific, Shanghai, China. Methanol was purchased from Sinopharm Chemical Reagent Co., Ltd., Shanghai, China. Acetonitrile was purchased from Chengdu Kelon Chemical Co., Ltd., Chendu, China. Ascorbic acid was obtained from Tianjin New Fine Chemical Development Center, Tianjin, China. Phenolphthalein was acquired from Shanghai Zhongqin Chemical Reagent Co., Ltd., Shanghai, China. The supplier of 3,5-Dinitrosalicylic acid was purchased from Zhengzhou Huiju Chemical Co., Ltd., Zhenzhou, China. Folin-phenol reagent was purchased from Tianjin Guangfu Technological Development Co., Ltd., Tianjin, China.

### 4.2. Apparatus

The following equipment was used: HPLC-FD (LC-20A, Thermo Fisher Scientific, Waltham, MA, USA), spectrophotometer (722N, Shanghai Yidian Analytical Instrument Co., Ltd., Shanghai, China), evaporator (RE-2000B, Shanghai Yarong Biochemical Instrument Factory, Shanghai, China), centrifuge (3K15, Beijing Wuzhou Oriental Technology Development Co., Ltd., Beijing, China), water bath (DK-98 IIA, Tianjin Taist Instrument Co., Ltd., Tianjin, China), high-pressure steam sterilizer (LDZX-50KBS, Shanghai Shen’an Medical Instrument Factory, Shanghai, China), and incubator (SPX-250-Z-S, Shanghai Yuejin Medical Instrument Co., Ltd., Shanghai, China).

### 4.3. Cultivation of Strains

The experiment was performed according to Guo’s method, and adjusted as required [57]. Potatoes were peeled and cleaned first and then cut into small pieces. About 200 g potato pieces were added to 1000 mL water and boiled for 30 min. The resulting mixture was filtered with a double-layer gauze, and 20 g of sucrose and agar was added to it. The mixture was also stirred and sterilized at a high temperature for 30 min. Then, *A. niger* spore suspension was added dropwise and cultured at 25 °C for 7 days. The cultures were flooded with 10 mL 0.05% Tween-80, and the cyst spores on the surface of the medium were gently scraped off. The concentration of *A. niger* (H1) spore suspension was adjusted to 1 × 10^6^ CFU/mL with a hemocytometer [58].

### 4.4. Injury Inoculation of Jujube Fruit

Jujubes were selected for uniformity (free from physical damage and disease). The jujubes were rinsed with tap water, sanitized in a 0.5% sodium hypochlorite solution for 2 min, and wiped on their surface with 75% ethanol. Then, a hole of similar size (diameter of about 2 mm, depth of about 8 mm) was punched on each fruit with a sterilized puncher. Next, the prepared *A. niger* (H1) spore suspension was inoculated into the well, and then the spore suspension was inoculated into the holes with a pipette gun. Finally, the inoculated fruit was sealed and cultured in a constant temperature incubator for 7 days.

### 4.5. Determination of Jujube Quality

#### 4.5.1. Weight Loss

The inoculated jujubes were weighed at 0, 3, 5, and 7 days by electronic balance. The results were calculated by the data obtained. The weight loss rate was measured according to the following formula:

Weight loss rate (%) = (M_0_ − M’)/M_0_ (1)

where M_0_: weight of day 0; M’: weight of different inoculation days (3, 5, 7)

#### 4.5.2. Reducing Sugar

Reducing sugar content was detected following the 3,5-Dinitrosalicylic acid (DNS) method of Zhang et al. (2018), with some modifications [59]. A total of 1 mL of jujube juice was mixed with 3 mL 3,5-Dinitrosalicylic acid, then centrifuged for 10 min at 6000× *g*. The reaction mix was further boiled at 100 °C for 6 min and cooled. The absorbance of the mixture was measured at 520 nm by a UV spectrophotometer. The amount of reducing sugar was obtained by comparing the absorbance of the glucose standard solution.

#### 4.5.3. Total Acid

A total of 10 g of jujube samples were weighed in a 100 mL volumetric flask, and sufficient distilled water was added, so that the final volume would be 100 mL. After filtration, 2 drops of 1% phenolphthalein were added to 20 mL filtrate, and the mixed solution was titrated with standard sodium hydroxide. This procedure was repeated three times. Therefore, the total acid content can be calculated by the amount of sodium hydroxide used. Total acid was measured according to the following formula:

(2) $$X=\frac{V}{W}\times \frac{C\times K\times V_{2}}{V_{1}}\times 100$$

where X: total acid (g/mL); V: volume after dilution (mL); V_1_: sample volume (mL); V_2_: sodium hydroxide volume (mL); C: sodium hydroxide concentration (moL/L); W: sample weight (g); K: conversion coefficient.

#### 4.5.4. Vitamin C

A total of 2 mL of 1% HCl was added to jujube powder, and the mixture was shaken. Homogenates were transferred to a 25 mL volumetric flask. Extracted solutions (0.1 mL) were mixed with 10% HCL (0.2 mL) in a 10 mL volumetric flask. The extinction value was measured at 243 nm, and distilled water was used as control. Otherwise, the extinction value of the solution could be measured, and the alkali treatment solution (0.1 mL extraction solution, 2 mL H_2_O, 0.6 mL 1 mol/L sodium hydroxide solution, and 0.6 mL 10% hydrochloric acid) was used as a control. Thus, the content of vitamin C was calculated through the standard curve.

#### 4.5.5. Total Phenol

Total phenol content was detected following the method of Zhang et al. (2018) with some modifications [60]. A series of gallic acid standard solutions (0, 12.5, 25, 50, 100, 200, and 400 mg/L) were prepared, respectively. A sample of 5 g of jujube powder was ground into powder and dissolved in distilled water. After the jujube sample was centrifuged for 10 min at 3000 r/min, the supernatant was taken and diluted 50 times. The reaction began when 1 mL of the standard solution and sample solution were added to a solution of 1 mL of Folin–Ciocalteu reagent and 3 mL of 20% Na_2_CO_3_ solution. The mixture was reacted in a water bath at 50 °C for 30 min. The absorbance at 765 nm was recorded. The total phenolic content was calculated by the regression equation between the absorbance A and the concentration of the gallic acid standard solution (mg/mL).

#### 4.5.6. Color Value

The color of the jujube fruit peel (exocarp) was measured using a colorimeter. The peel color was recorded using the L* value, a* value, and b* value of the middle part of the jujube. Three points on each jujube were measured.

#### 4.5.7. Lesion Diameter

In this experiment, the diameter of the lesion was measured according to Chang’s method [61]. The lesion diameter is the mean number of the sum of transverse and longitudinal diameters of diseased spots of the jujube. Each treatment was repeated three times, and was used to assess disease severity once every 2 d.

### 4.6. Sample Extraction

A total of 5 g of jujube was mixed with 10 mL of 84:16 (*v*/*v*) acetonitrile/water. The mixture was centrifuged at 5000 r/min for 5 min, then filtered to 7 mL. The solution was purified with an MFC229 multifunctional solid phase purification column (Pribolab, Qingdao, China). The eluate was evaporated to dryness under a nitrogen stream, and then 1 mL mobile phase acetonitrile/water (1/1, *v*/*v*) was used for vortex mixing for 30 s and passed through a filter (0.22 µm) for the determination of OTA concentration.

### 4.7. Standard Curve

The 0.1 mg/mL OTA standard solution was diluted with acetonitrile/water solvent (84:16) to obtain a series of concentrations (0.5–50 ng/mL) of OTA. High-performance liquid chromatograph-fluorescence detection (HPLC-FD) was applied to study the absorption peak changes in different concentrations of OTA.

### 4.8. HPLC Detection

High-performance liquid chromatography (HPLC) with a fluorescence detector was used to quantify the analyte. The column used was a ZORBAX Eclipse Plus C18 column (4.6 mm × 250 mm × 5 µm). The column temperature was 35 °C; the injection volume was 50 μL; the flow rate was 1.0 mL/min; and the mobile phase was acetonitrile/water (1:1, *v*/*v*). The fluorescence detection conditions were as follows: the excitation wavelength was 333 nm, and the emission wavelength was 460 nm [58].

### 4.9. Statistical Analysis

Results were presented as mean value ± standard errors; statistical analysis was performed with SPSS version 19.0. To determine the effect of treatments, the data were compared with a Duncan test. Differences at *p* < 0.05 were considered as significant. Figures were drawn in Origin 2018.

## Figures and Tables

**Table 1:** Changes in the weight loss of jujubes before and after infection with *A. niger* (H1). All data are expressed as means ± standard deviation of triplicate samples. Different lower-case letters indicate significant differences according to Duncan’s test (*p* < 0.05).

Days	Treatment (g)	Control (g)	Treatment (%)	Control (%)
0	12.60 ± 0.14 ^b^	10.50 ± 0.46 ^b^	0.00 ± 0.00 ^a^	0.00 ± 0.00 ^a^
3	12.24 ± 0.10 ^b^	9.78 ± 0.36 ^b^	0.03 ± 0.01 ^a^	0.07 ± 0.14 ^a,b^
5	12.16 ± 0.18 ^b^	9.46 ± 0.38 ^b^	0.03 ± 0.01 ^a^	0.08 ± 0.15 ^a,b^
7	10.76 ± 0.29 ^a^	7.43 ± 1.26 ^a^	0.15 ± 0.03 ^b^	0.29 ± 0.15 ^b^

## Data Availability

The data presented in this study are included in the article; further inquiries can be directed to the corresponding author.

## References

[1] Liu M.J., Zhao J.C., Cai Q.L., Liu G.C., Wang J.R., Zhao Z.H., Liu P.D., Dai L., Yan G.J., Wang W.J. (2014). The complex jujube genome provides insights into fruit tree biology. Nat. Commun..

[2] Ji X.L., Hou C.Y., Yan Y.Z., Shi M.M., Liu Y.Q. (2020). Comparison of structural characterization and antioxidant activity of polysaccharides from jujube (*Ziziphus jujuba* Mill.) fruit. Int. J. Biol. Macromol..

[3] Liu M.J., Wang J.R., Liu P., Zhao Z.H., Dai L., Li X.S., Liu Z.G. (2015). Historical achievements and frontier advances in the production and research of Chinese jujube (*Ziziphus jujuba* Mill.) in China. Acta Hortic. Sin..

[4] Liu M.J., Wang J.R. (2019). Fruit scientific research in new China in the past 70 years: Chinese jujube. J. Fruit Sci..

[5] Krska B., Mishra S., Liu M.J. (2009). Sensory evaluation of different products of *Ziziphus Jujuba* Mill. Acta Hortic..

[6] Liu M.J., Wang J.R., Wang L.L., Liu P., Zhao J., Zhao Z.H., Yao S.R., Florin S., Liu Z.G., Wang L.X. (2020). The historical and current research progress on jujube–a superfruit for the future. Hortic. Res..

[7] Jiang J.G., Huang X.J., Chen J., Lin Q.S. (2007). Comparison of the sedative and hypnotic effects of flavonoids, saponins, and polysaccharides extracted from Semen. Ziziphus jujube. Nat. Prod. Res..

[8] Zhao L.W., Zhang L., Liu F.M., Xue X.F., Pan C.P. (2014). Multiresidue analysis of 16 pesticides in jujube using gas chromatography and mass spectrometry with multiwalled carbon nanotubes as a sorbent. J. Sep. Sci..

[9] Song L.H., Meinhardt L.W., Bailey B., Zhang D. (2019). Genetic improvement of Chinese jujube for disease resistances: Status, knowledge gaps and research needs. Crop Breed. Genet. Genom..

[10] Baker S.E. (2006). *Aspergillus niger* genomics: Past, present and into the future. Med. Mycol..

[11] Massi F.P., Iamanaka B.T., Barbosa R.L., Sartori D., Ferrrant L., Taniwaki M.H., Fungar M.H.P. (2020). Molecular analysis of *Aspergillus* section *Nigri* isolated from onion samples reveals the prevalence of A. welwitschia. Braz. J. Microbiol..

[12] Chen Y. (2020). Nitric Oxide Fumigation on Disease Inhibition and Mycotoxin Removal in Dried Fruit During Storage. Ph.D. Thesis.

[13] Zhang G.H., Wang L., Zhao M.F., He Y.Q. (2006). Investigation on main fungal diseases of maoye jujube in Yunnan Province. Plant Prot..

[14] Guo D.Q., Chen H.X., Song Y.T., Zhu L.X. (2015). Study on bioloy of Yuancui jujube after harvest infected by *Aspergillus niger* TL-10 and its control. China Plant Prot..

[15] Chen Y., Zhang J., Wei J., Wu B., Zhang C., Wang J.D. (2019). Nitric oxide fumigation inhibiting Apgliluss niger disease and maintaining storage quality of dried ash jujube. Trans. Chin. Soc. Agric. Eng..

[16] Klingelhofer D., Braun M., Schoffel N., Oremek G.M., Bruggmann D., Groneberg D.A. (2020). Ochratoxin-Characteristics, influences and challenges of global research. Food Control.

[17] Nan M.N., Xue H.L., Bi Y. (2022). Contamination, Detection and Control of Mycotoxins in Fruits and Vegetables. Toxins.

[18] Sage L., Krivobok S., Delbos E., Seigle-Murandi F., Creppy E.E. (2002). Fungal flora and ochratoxin A production in grapes and musts from France. J. Agric. Food Chem..

[19] Rahimi E., Shakerian A. (2013). Ochratoxin A in dried figs, raisings, apricots, dates on Iranian retail market. Health.

[20] Iamanaka B.T., Taniwaki M.H., Menezes H.C., Vicente E., Fungaro M.H.P. (2005). Incidence of toxigenic fungi and ochratoxin A in dried fruits sold in Brazil. Food Addit. Contam..

[21] Azaiez I., Font G., Mañes J., Fernández-Franzón M. (2015). Survey of mycotoxins in dates and dried fruits from Tunisian and Spanish markets. Food Control.

[22] Zhang X.X., Ou X.Q., Zhou Z.Y., Ma L.Y. (2015). Ochratoxin A in Chinese dried jujube: Method development and survey. Food. Addit. Contam. Part A.

[23] Zheng Y.L., Jia X.Y., Ran Y.L., Du M.J., Zhao Z.Y., Chen L., Zhang P., Li J.K., Yuan J.W., Wang H.F. (2022). Inhibition effect of *Aspergillus niger* and quality preservation of apple by in-package sterilization medium flow of circulating. Sci. Hortic..

[24] Li L.M., Liu X., Li X.H., Yang H.Y., Zhao Y.T. (2020). Study on the Wound Pahogenciy of *Aspergills niger* and lts Antonistic Bacteria Sreening of Postharvest Red Globe Grapes at Room Tmperature. Food Res. Dev..

[25] Birgitte N.B., Leif P. (2000). Influence of arbuscular mycorrhizal fungi on soil structure and aggregate stability of a vertisol. Plant. Soil..

[26] Zhang L., Wei J., Zhang Z., Wu B. (2018). Effect of Gaseous H2S Fumigation on Aspergillusnige Inhibition and Posthar-vest Quality of Table Grape. Mod. Food Sci. Technol..

[27] Zhao Z.H., Liu M.J., Tu P.F. (2008). Characterization of water soluble polysaccharides from organs of Chinese Jujube (*Ziziphus jujuba* Mill. cv. Dongzao). Eur. Food Res. Technol..

[28] Morkunas I., Ratajczak L. (2014). The role of sugar signaling in plant defense responses against fungal pathogens. Acta Physiol. Plant..

[29] Wang S.S., Han Y.H., Chen J.L., Zhang D.C., Shi X.X., Ye Y.X., Chen D.L., Li M. (2018). Insights into Bacterial Cellulose Biosynthesis from Different Carbon Sources and the Associated Biochemical Transformation Pathways in Komagataeibacter sp W1. Polymers.

[30] Lin Y.Z., Li N., Lin H.T., Lin M.S., Chen Y.H., Wang H., Ritenour M.A., Lin Y.F. (2020). Effects of chitosan treatment on the storability and quality properties of longan fruit during storage. Food Chem..

[31] Lei A.Q., Liu Q.S., Li Y., Zou Y.N., Wu Q.S. (2022). Effects of symbiotic fungi on fruit quality and soil characteristics of Lane Late navel orange. J. Huazhong Agric. Univ..

[32] Wei S., Hu C.J., Nie P., Zhai H.C., Zhang S.B., Li N., Lv Y.Y., Hu Y.S. (2022). Insights into the Underlying Mechanism of Ochratoxin A Production in *Aspergillus niger* CBS 513.88 Using Different Carbon Sources. Toxins.

[33] Fan Y.Y., Hu D.Q., Zhang R.L., Li X.L., He W.Z., Hua W.Z., Li J., Wu A.B., Wang C. (2020). Effects of Black Spot Disease on Nutritional Composition of Red Jujubes Grown in Xinjiang. Food Sci..

[34] Liu F., Tan Y., Wang Y., Wu T., Zhang X.Z. (2015). Change of Disease Related Sub-stances Content in Branches During the Process of Valsa ceratosperma. Chin. Agric. Sci. Bull..

[35] (2009). The Physiological and Molecular [w] Responses of Arabidopsis thaliana to the Stress of Oxalic Acid. Agric. Sci. China.

[36] Hua S.H., Hsu H.C., Han P. (2019). Development of Detection System with Low Predictive Errors for Determining Vitamin C Content of Indian Jujube. Appl. Sci..

[37] Claudio S., Edward C.Y. (2001). Ascorbic acid metabolism during white spruce somatic embryo maturation and germination. Physiol. Plant..

[38] Tabata K., Takaoka T., Esaka M. (2002). Gene expression of ascorbic acid-related enzymes in tobacco. Phytochemistry.

[39] Lang C., Dörnenburg H. (2000). Perspectives in the biological function and the technological application of polygalacturonases. Appl. Microbiol. Biot..

[40] Tang X.L., Liu H.G., Xiao Y., Wu L., Shu P. (2022). Vitamin C Intake and Ischemic Stroke. Front. Nutr..

[41] Xu W.Y. (2020). Effect of Umbelliferone Tteatment on Fruit Quality and Disease Resistance of “XuXiang” Kiwifrutt. Master’s Thesis.

[42] Cao S.F., Shao J.R., Shi L.Y., Xu L.W., Shen Z.M., Chen W., Yang Z.F. (2018). Melatonin increases chilling tolerance in postharvest peach fruit by alleviating oxidative damage. Sci. Rep..

[43] Liu J., Liu X., Bi J.F., Wu X.Y., Zhou L.Y., Ruan W.H., Zhou M., Jiao Y. (2018). Kinetic modeling of non-enzymatic browning and changes of physio-chemical parameters of peach juice during storage. J. Food. Sci. Technol..

[44] Liao Y., Yin L.G., Si C.J., Tie Y., Jiang Y.D., Zhao J.Y. (2023). Effects of solid fermentation of *Aspergillus*, yeast and lactic acid bacteria on antioxidant activity of polyphenols and flavonoids in Figure. Southwest China J. Agric. Sci..

[45] Ajila C.M., Gassara F., Brar S.K., Verma M., Tyagi R.D., Valéro J.R. (2012). Polyphenolic antioxidant mobilization in apple pomace by different methods of solid-state fermentation and evaluation of its antioxidant activity. Food Bioprocess. Technol..

[46] Chen H., Sun Z., Yang H. (2019). Effect of carnauba wax-based coating containing glycerol monolaurate on the quality maintenance and shelf-life of Indian jujube (*Zizyphus mauritiana* Lamk.) fruit during storage. Sci. Hortic..

[47] Stanic-Vucinic D., Prodic I., Apostolovic D., Nikolic M., Velickovic T.C. (2013). Structure and antioxidant activity of β-lactoglobulin-glycoconjugates obtained by high-intensity-ultrasound-induced Maillard reaction in aqueous model systems under neutral conditions. Food Chem..

[48] Tian Y. (2020). Effect of *Aspergillus niger* on Southern-Route Tea Quality during Fermentation. Bachelor’s Thesis.

[49] Chandini S.K., Rao L.J., Gowthaman M.K., Haware D.J., Subramanian R. (2011). Enzymatic treatment to improve the quality of black tea extracts. Food Chem..

[50] Zhang J., Wu X., Zhang S.Q. (2008). Antifungal mechanism of antibacterial peptide, ABP-CM4, from Bombyx mori against *Aspergillus niger*. Biotechnol. Lett..

[51] Zhang H.H., Wang Y., Zhang X.L., Wang J. (2017). An ochratoxigenic strain of *Aspergillus niger* and its ochratoxin A production conditions. Mycosystema.

[52] He Y.L., Liang Z.H., Xu W.T., Huang K.L., Luo Y.B. (2009). Study on the isolation of *Aspergilus* carbonarius and ochratoxin A production from wine grape. Sino-Overseas Grapevine Wine.

[53] Gómez C., Bragulat M.R., Abarca M.L., Mínguez S., Cabañes F.J. (2006). Ochratoxin A-producing fungi from grapes intended for liqueur wine production. Food Microbiol..

[54] Abrunhosa L., Serra R., Venancio A. (2002). Biodegradation of ochratoxin A by fungi isolated from grapes. J. Agric. Food Chem..

[55] Varga J., Rigó K., Téren J. (2000). Degradation of ochratoxin A by *Aspergillus* species. Int. J. Food Microbiol..

[56] Bejaoui H., Mathieu F., Taillandier P., Lebrihi A. (2006). Biodegradation of ochratoxin A by *Aspergillus* section *Nigri* species isolated from French grapes: A potential means of ochratoxin A decontamination in grape juices and musts. FEMS Microbiol. Lett..

[57] Guo D., Zhu L., Hou X. (2015). Combination of UV-C Treatment and *Metschnikowia pulcherrimas* for Controlling *Alternaria* Rot in Postharvest Winter Jujube Fruit. J. Food Sci..

[58] Nan M.N., Bi Y., Xue H.L., Xue S.L., Long H.T., Pu L.M., Fu G.R. (2019). Rapid determination of ochratoxin A in grape and its commodities based on a label-free impedimetric aptasensor constructed by layer-by-layer self-assembly. Toxins.

[59] Zhang L.H., Zha M.M., Li S.F., Zong W. (2022). Investigation on the effect of thermal sterilization versus non-thermal sterilization on the quality parameters of jujube juice fermented by Lactobacillus plantarum. J. Food Sci. Technol..

[60] Liu C.Q., Wang Q.L., Feng X.J., Zhang P., Xi G.C., Wang Y.N., Zhen W.C., Chen D., Cang K.D., Cao M. (2009). Study on the antioxidants and total phenol content of optimally selected jujube cultivars. Acta Hortic..

[61] Chang L.L., Yu Y.W., Zhang L.L., Wang X.J., Zhang S.Y. (2022). Arginine induces the resistance of postharvest jujube fruit against Alternaria rot. Food Qual. Saf..

